# Supplementary material for: Modeling glioblastoma heterogeneity as a dynamic network of cell states
Source: Mol Syst Biol. 2021 Sep 16;17(9):e10105. doi: 10.15252/msb.202010105 (PMC8444284; doi:10.15252/msb.202010105)
Supplement: Supplementary file 5 — Source Data for Figure 3 [file MSB-17-e10105-s001.zip › Figure3A_sourcedata/GSEA_3065/nosigdb_state3.GseaPreranked.1622718426028/RICHARDS_VERHAAK_CANCERCELL_2010_NEURAL.html]

Details for gene set RICHARDS\_VERHAAK\_CANCERCELL\_2010\_NEURAL[GSEA]

|  || Dataset | state3 |
| Phenotype | NoPhenotypeAvailable |
| Upregulated in class | na\_neg |
| GeneSet | RICHARDS\_VERHAAK\_CANCERCELL\_2010\_NEURAL |
| Enrichment Score (ES) | -0.52051336 |
| Normalized Enrichment Score (NES) | -1.7285559 |
| Nominal p-value | 0.008116883 |
| FDR q-value | 0.008891712 |
| FWER p-Value | 0.145 |
Table: GSEA Results Summary

  

Fig 1: Enrichment plot: RICHARDS\_VERHAAK\_CANCERCELL\_2010\_NEURAL      
 Profile of the Running ES Score & Positions of GeneSet Members on the Rank Ordered List

  

| PROBE | GENE SYMBOL | GENE\_TITLE | RANK IN GENE LIST | RANK METRIC SCORE | RUNNING ES | CORE ENRICHMENT || 1 | SAR1A |  |  | 1295 | 0.116 | -0.1235 | No |
| 2 | USP33 |  |  | 1537 | 0.101 | -0.1289 | No |
| 3 | TSNAX |  |  | 2090 | 0.072 | -0.1765 | No |
| 4 | SPAST |  |  | 2560 | 0.055 | -0.2183 | No |
| 5 | ANKRD46 |  |  | 2825 | 0.046 | -0.2385 | No |
| 6 | FHIT |  |  | 2853 | 0.046 | -0.2315 | No |
| 7 | ADD3 |  |  | 2929 | 0.043 | -0.2307 | No |
| 8 | CAMK2G |  |  | 3210 | 0.035 | -0.2551 | No |
| 9 | SHTN1 |  |  | 3302 | 0.033 | -0.2582 | No |
| 10 | IMPA1 |  |  | 3375 | 0.031 | -0.2596 | No |
| 11 | MAGEH1 |  |  | 3491 | 0.028 | -0.2666 | No |
| 12 | SNX11 |  |  | 4037 | 0.014 | -0.3262 | No |
| 13 | SCHIP1 |  |  | 4109 | 0.012 | -0.3316 | No |
| 14 | FAM49B |  |  | 4184 | 0.010 | -0.3379 | No |
| 15 | PPA1 |  |  | 4255 | 0.008 | -0.3442 | No |
| 16 | CCK |  |  | 4463 | 0.002 | -0.3675 | No |
| 17 | FBXO3 |  |  | 4477 | 0.002 | -0.3686 | No |
| 18 | ANXA7 |  |  | 4553 | -0.000 | -0.3772 | No |
| 19 | MDH1 |  |  | 4655 | -0.003 | -0.3882 | No |
| 20 | AGTPBP1 |  |  | 4724 | -0.004 | -0.3951 | No |
| 21 | CRBN |  |  | 4987 | -0.010 | -0.4230 | No |
| 22 | PPP2R5A |  |  | 5068 | -0.013 | -0.4294 | No |
| 23 | GNAI1 |  |  | 5194 | -0.016 | -0.4403 | No |
| 24 | ORC4 |  |  | 5213 | -0.016 | -0.4388 | No |
| 25 | ROGDI |  |  | 5266 | -0.017 | -0.4410 | No |
| 26 | MGST3 |  |  | 5395 | -0.021 | -0.4512 | No |
| 27 | CRYL1 |  |  | 5540 | -0.025 | -0.4621 | No |
| 28 | CRYZL1 |  |  | 5689 | -0.030 | -0.4726 | No |
| 29 | ACSL4 |  |  | 5729 | -0.031 | -0.4703 | No |
| 30 | PEX19 |  |  | 5768 | -0.032 | -0.4677 | No |
| 31 | MAT2B |  |  | 5925 | -0.037 | -0.4776 | No |
| 32 | CUTC |  |  | 5984 | -0.039 | -0.4757 | No |
| 33 | MORF4L2 |  |  | 6043 | -0.041 | -0.4733 | No |
| 34 | NSL1 |  |  | 6090 | -0.042 | -0.4693 | No |
| 35 | THTPA |  |  | 6160 | -0.045 | -0.4673 | No |
| 36 | TCEAL1 |  |  | 6281 | -0.049 | -0.4703 | No |
| 37 | MRPL49 |  |  | 6719 | -0.067 | -0.5058 | Yes |
| 38 | NDUFS3 |  |  | 6821 | -0.072 | -0.5016 | Yes |
| 39 | ACYP2 |  |  | 6848 | -0.073 | -0.4884 | Yes |
| 40 | LYRM1 |  |  | 6853 | -0.073 | -0.4727 | Yes |
| 41 | PRPSAP2 |  |  | 6890 | -0.075 | -0.4602 | Yes |
| 42 | CDC42 |  |  | 7126 | -0.089 | -0.4677 | Yes |
| 43 | MSRB2 |  |  | 7268 | -0.098 | -0.4623 | Yes |
| 44 | TTC1 |  |  | 7379 | -0.107 | -0.4515 | Yes |
| 45 | PDE6D |  |  | 7435 | -0.111 | -0.4333 | Yes |
| 46 | YPEL5 |  |  | 7616 | -0.129 | -0.4255 | Yes |
| 47 | CHN1 |  |  | 7653 | -0.133 | -0.4004 | Yes |
| 48 | HPRT1 |  |  | 7679 | -0.137 | -0.3732 | Yes |
| 49 | ANKS1B |  |  | 7710 | -0.141 | -0.3456 | Yes |
| 50 | TPM3 |  |  | 7943 | -0.175 | -0.3337 | Yes |
| 51 | POPDC3 |  |  | 8033 | -0.190 | -0.3021 | Yes |
| 52 | UROS |  |  | 8099 | -0.203 | -0.2648 | Yes |
| 53 | CALM2 |  |  | 8295 | -0.257 | -0.2306 | Yes |
| 54 | GABARAPL2 |  |  | 8419 | -0.301 | -0.1784 | Yes |
| 55 | COX5B |  |  | 8448 | -0.314 | -0.1124 | Yes |
| 56 | GUK1 |  |  | 8738 | -0.668 | 0.0015 | Yes |
Table: GSEA details [plain text format]

  

Fig 2: RICHARDS\_VERHAAK\_CANCERCELL\_2010\_NEURAL: Random ES distribution      
 Gene set null distribution of ES for **RICHARDS\_VERHAAK\_CANCERCELL\_2010\_NEURAL**
